# 儿童异基因造血干细胞移植后供者HLA识别巨细胞病毒抗原肽亲和力与患者巨细胞病毒感染的相关性

**DOI:** 10.3760/cma.j.issn.0253-2727.2021.09.008

**Published:** 2021-09

**Authors:** 李 杨, 卓 王, 沙 吴, 文婕 卢, 昊 熊

**Affiliations:** 华中科技大学同济医学院附属武汉儿童医院血液肿瘤科 430016 Department of Hematology and oncology, Wuhan children's Hospital Affiliated to Tongji Medical College, Huazhong University of science and technology, Wuhan 430016, China

**Keywords:** 异基因造血干细胞移植, 人类白细胞抗原, 巨细胞病毒, 儿童, Allogeneic hematopoietic stem cell transplantation, Human leukocyte antigen, Cytomegalovirus, Children

## Abstract

**目的:**

探索儿童异基因造血干细胞移植（allo-HSCT）后供者HLA型别识别不同巨细胞病毒（CMV）抗原肽亲和力与发生CMV感染的相关性。

**方法:**

回顾性分析武汉儿童医院血液肿瘤科146例allo-HSCT患儿移植后6个月内发生CMV再激活、CMV感染、CMV组织器官病的数据，分析其与供者HLA型别识别CMV抗原肽差异的相关性。

**结果:**

纳入研究的146例移植后患儿中HLA型别与PP65单独高亲和力82例（56.16％），移植后发生CMV感染34例（41.46％）；IE-1高亲和力型5例，移植后感染0例；无明确亲和力多肽2例，感染0例；PP65+IE-1高亲和力型52例，移植后感染13例（25.00％）；与PP65和PP50共同高亲和力型5例，移植后感染3例；未遇到仅与PP50高亲和力HLA供者。与仅携带PP65高亲和力等位基因组比较，伴有IE-1高亲和力HLA位点供者移植后表现出更低的CMV感染发生率（22.81％对41.46％，*P*＝0.029）。

**结论:**

CMV PP65和IE-1高亲和力型别HLA类型覆盖约99.8％的供者，选择CMV抗原肽IE-1高亲和力型别HLA供者或可降低儿童血液病患者allo-HSCT后CMV感染风险。

## Abstract (en)

**Objective:**

To explore the correlation between the affinity while donor's HLA type recognizing different cytomegalovirus (CMV) antigen peptide and the occurrence of CMV infection after allogeneic hematopoietic stem cell transplantation (allo-HSCT) in children.

**Methods:**

To investigate the relationship between CMV reactivation, CMV infection or CMV related tissue/organ diseases and the different HLA-type recognition antigen peptide of the donors, we retrospectively analyzed the clinical data of 146 children with CMV infection for 6 months since from the time they underwent transplantation in Wuhan Children's Hospital.

**Results:**

Among 146 patients, the HLA type of 82 (56.16％) cases had high affinity with PP65 alone, and 34 cases of CMV infection occurred after transplantation (41.46％). None of 5 cases that had a high affinity with IE-1 alone got CMV infection. None of 2 cases with no clear high-affinity peptide had CMV infection. Three of 5 cases that had a high affinity with PP65 and PP50 had CMV infection. Thirteen of 52 cases that had a high affinity with PP65 and IE-1 had CMV infection (25.00％). HLA with exclusive PP50 high affinity was not encountered. Donors with a high-affinity HLA locus associated with IE-1 showed a lower incidence of CMV infection after HSCT compared to those carrying only the PP65 high-affinity allele (22.81％ *vs* 41.46％, *P*＝0.029).

**Conclusion:**

HLA type with PP65 and IE-1 high-affinity covers approximately 99.8％ of the donors. Stem cells generated from HLA donors with high affinity with the CMV antigen peptide IE-1 can reduce the risk of post-transplantation CMV-activated infection in children.

异基因造血干细胞移植（allo-HSCT）是儿童高危血液肿瘤及多种先天性免疫遗传缺陷性病治愈的唯一手段[1]。移植后巨细胞病毒（CMV）再激活在allo-HSCT患者中的发生率约为60％，是导致移植失败和死亡的重要原因之一[2]。人类白细胞抗原（HLA）又称主要组织相容性复合体（MHC），不仅在儿童造血干细胞移植供者的选择、移植后植入、移植物抗宿主病（GVHD）等方面具有重要作用，新近发现其也与免疫细胞识别CMV抗原肽和抗病毒免疫存在密切关系，不同HLA型别个体免疫细胞对CMV主要抗原肽如PP65、IE-1等具有不同的亲和力和不同的免疫反应效应[3]–[4]。以往研究发现，用不同抗原肽监测移植后CMV特异性免疫细胞斑点数量具有潜在的临床预测价值[5]–[6]，但供者不同HLA表型识别抗原肽亲和力不同与儿童allo-HSCT后CMV激活感染关系尚未见明确报道。本研究分析在我院接受allo-HSCT的146例儿童移植后CMV感染发生的临床特性，以期探索移植后CMV再激活与供者HLA型别的相关性。

## 病例与方法

1. 病例资料：2016年8月至2020年5月在武汉儿童医院血液肿瘤科接受allo-HSCT的146例患儿纳入本研究。均为外周血造血干细胞移植，移植前供者患者外周血CMV-DNA拷贝数均小于1×10^3^/L。

2. 预处理及GVHD预防方案：94例采用以白消安（Bu）+环磷酰胺（CTX）+兔抗人胸腺细胞免疫球蛋白（rATG，即复宁^®^）为基础的预处理方案（Bu 0.8～1.2 mg/kg每6 h 1次×4 d；CTX 50 mg·kg^−1^·d^−1^×4 d；rATG总量5～10 mg/kg，分3 d给药）；52例采用后置环磷酰胺（PT-Cy）方案（CTX 10 mg·kg^−1^·d^−1^，−8 d、−7 d；CTX 50 mg·kg^−1^·d^−1^，+3 d、+4 d；司莫司汀250 mg/m^2^，−6 d；Bu 0.8 mg/kg每6 h 1次，−5 d～−2 d）；氟达拉滨（Flu）40 mg·m^−2^ ·d^−1^，−6 d～−2 d；rATG 1 mg·kg^−1^·d^−1^，+5 d、+6 d。

Bu-Cy方案预处理患者均以霉酚酸酯（MMF）+环孢素A（CsA）+短程甲氨蝶呤作为GVHD预防方案；PT-Cy方案患儿造血干细胞回输后72 h给予CTX 50 mg·kg^−1^·d^−1^连用2 d，CsA和MMF于造血干细胞回输后第5天开始，CsA 3 mg·kg^−1^·d^−1^，至患儿造血重建且胃肠功能恢复后改为口服，维持全血谷浓度150～250 mg/L；MMF 30 mg·kg^−1^ ·d^−1^口服；rATG 1 mg·kg^−1^ ·d^−1^，+5、+6 d，若无GVHD，MMF于+30 d剂量减半，+45 d停用。

3. 常规感染预防措施：患者于预处理开始至粒细胞植活期间入住层流室内，并施以反向隔离措施。口服复方磺胺甲基异恶唑预防肺孢子菌病；应用棘白霉素（米卡芬净或卡泊芬净）预防真菌感染；静脉更昔洛韦预防单纯疱疹、水痘-带状疱疹病毒及CMV感染（−10 d～−1 d）。

4. CMV监测：移植前供受者均常规检测CMV IgG/IgM抗体及CMV-DNA，移植后每周检测1次全血CMV-DNA，若CMV-DNA拷贝数>5×10^2^/L则每周检测2次。CMV组织器官病的诊断标准参照文献[7]。CMV肺炎确诊标准：①气促、胸闷等临床症状；②影像学检查发现多发浸润病灶；③肺泡灌洗液检测CMV-DNA拷贝数>1×10^3^/L。CMV肠炎确诊标准：①恶心、呕吐、腹痛、腹泻等临床症状；②肠镜活检病损部位黏膜CMV病原学检查阳性。CMV脑炎确诊标准：①临床中枢神经系统症状；②影像学检查发现颅内多发浸润病灶；③实验室脑脊液NGS检测CMV-DNA拷贝数>1×10^3^/L。CMV视网膜炎诊断标准：①视力下降等临床症状；②眼底出现炎性肿胀或出血渗出；③房水CMV-DNA拷贝数>1×10^3^/L。

5. CMV治疗方案：①预处理期间使用更昔洛韦预防（10 mg·kg^−1^·d^−1^静脉滴注，−10 d～−1 d）。②监测外周血CMV-DNA，前6个月每周1～2次，以后至移植后1年每2周1次（免疫抑制治疗时恢复监测）。③CMV-DNA检测1次阳性者（>5×10^2^/L），应用更昔洛韦（5 mg/kg每12 h 1次）或膦甲酸钠（90～120 mg·kg^−1^·d^−1^）（造血重建差者首选膦甲酸钠）。④一线治疗10～14 d后CMV-DNA拷贝数不下降或任意时间较初始CMV-DNA拷贝数上升2个对数级，则考虑联合抗病毒治疗。若治疗2周CMV-DNA不转阴，考虑输注CMV特异性细胞毒性T细胞（CMV-CTL）。CMV-DNA连续2次阴性改为口服缬更昔洛韦降阶梯治疗2周，如CMV-DNA持续阴性，改为阿昔洛韦口服。

6. 随访：采用查阅住院、门诊病历或电话随访方式获取+180 d内患者CMV感染情况。

7. HLA型别分组：根据文献报道的不同HLA等位基因识别CMV高亲和力抗原肽段不同将HLA结果分为PP65高亲和力组、IE-1高亲和力组、PP50高亲和力组、PP65+IE-1组、PP65+PP50组及未明确组。其中PP65高亲和力位点为HLA-A（02:01,24:02,02:07,11:01）、HLA-B（07:02,15:01, 40:02,40:06,44:03,51:01,40:01）、HLA-C（01:02,04:01,08:01,12:02,15:02）。IE-1高亲和力位点为HLA-A（02:01）、HLA-B（08:01）、HLA-C（07:02）。PP50高亲和力位点为HLA-A（01:01）。

8. 统计学处理：应用SPSS21.0数据统计包，采用秩和检验分析年龄构成比例；列联表资料分析数据，R×C表格资料卡方检验比较各组与总体之间的差异，四格表资料卡方检验比较两组之间的差异，二元logistic回归分析移植后CMV感染的危险因素，*P*<0.05为差异有统计学意义。

## 结果

1. 临床特征分析：146例患者中位年龄为79（9～183）个月，男87例，女59例。具体病种及移植类型和预处理方案在不同HLA型别中的分布见表1。

2. CMV感染的可能相关危险因素分析：如表1所示，卡方检验结果显示在不同HLA型别组中，供者来源、HLA相合程度、预处理方式差异均无统计学意义。在儿童allo-HSCT患者中，HLA相合程度、预处理方式、干细胞来源、预处理过程中ATG应用可能对移植后CMV再激活产生影响。在本研究中，考虑到所有患者均采用外周血干细胞移植，两种预处理方案均包含ATG，但在两种方案中ATG用量和GVHD预防方案有差异，故在CMV激活和无激活患者中进一步进行了两种预处理方式及HLA相合度（全相合与不全相合）的二分类Logistic回归分析，结果差异未见统计学意义（预处理方式：*P*＝0.817；HLA相合度：*P*＝0.070）。

**表1:** 146例异基因造血干细胞移植（allo-HSCT）患儿的临床特征

临床特征	PP65（82例）	IE-1（5例）	PP65+IE-1（52例）	PP65+PP50（5例）	未明确（2例）	*P*值
年龄［月，*M*（范围）］	36（1～156）	48（6～72）	48（3～108）	48（8～156）	30（12～48）	0.406
性别（例，男/女）	46/36	3/2	30/22	3/2	2/0	0.814
疾病类型（例）						
急性髓系白血病	18	0	14	2	1	0.484
幼年性粒-单核细胞白血病	3	0	1	0	0	0.948
骨髓增生异常综合征	5	0	3	0	0	0.943
急性淋巴细胞白血病	6	2	5	0	0	0.140
再生障碍性贫血	28	1	18	2	1	0.945
急性混合白血病	1	1	0	1	0	1.000
地中海贫血	7	1	1	0	0	0.339
范可尼贫血	2	0	2	0	0	0.963
黏多糖贮积症	3	0	1	0	0	0.945
噬血细胞综合征	2	0	6	0	0	0.198
先天性中性粒细胞缺乏	1	0	0	0	0	0.940
脑白质营养不良	1	0	1	0	0	0.990
WAS综合征	2	0	0	0	0	0.819
X-连锁淋巴增生综合征	1	0	0	0	0	0.940
MPIG6B基因突变	1	0	0	0	0	0.940
高IgM血症	1	0	0	0	0	0.940
供者类型（例）						
单倍型	44	2	34	3	1	0.644
同胞全相合	10	2	3	1	1	0.064
无关全相合	17	0	6	0	0	0.346
无关不全相合	11	1	9	1	0	0.514
预处理方案（例）						
Bu-Cy+rATG	59	4	27	2	2	0.075
PT-Cy+rATG	23	1	25	3	0	0.075

注：Bu-Cy：白消安+环磷酰胺；PT-Cy：移植后环磷酰胺；rATG：兔抗人胸腺细胞球蛋白（即复宁^®^）

3. 供者HLA型别结合CMV抗原肽亲和力分布：146例供者HLA配型结果显示，137例（93.84％）携带PP65高亲和力等位基因位点，57例（39.04％）携带IE-1高亲和力等位基因位点，5例（0.03％）携带PP50高亲和力位点并同时伴有PP65高亲和力位点，2例（0.01％）未见明确报道高亲和力抗原肽型别。

4. 移植后6个月内CMV感染情况：146例患者移植后6个月内发生CMV激活感染者51例（总体感染率34.93％），携带PP65高亲和力等位基因139例中51例（36.69％）发生感染，携带IE-1高亲和力等位基因57例中13例（22.81％）发生感染，单独携带PP65高亲和力等位基因组（不伴PP50或IE-1）82例中34例（41.46％）发生感染，单独携带IE-1高亲和力等位基因组5例均未发生感染，同时携带PP65+PP50高亲和力位点5例中3例发生感染。上述结果提示PP65高亲和力等位基因在移植后CMV感染免疫中不具有保护作用，而IE-1高亲和力HLA型别可能具有一定的抗感染优势。在57例携带IE-1高亲和力等位基因供者中52例伴有PP65高亲和力等位基因，将此57例供者与82例仅携带PP65高亲和力等位基因者进行比较，两组移植后CMV感染率分别为22.81％、41.46％（*P*＝0.029）。具体见表2。

**表2:** 不同HLA型别组异基因造血干细胞移植后巨细胞病毒（CMV）感染发生率

HLA型别分组	例数	CMV感染［例（％）］	*P*值（*P*_1_：与总体比较；*P*_2_：与单独PP65组比较）
总体	146	51（34.93）	
单独PP65	82	34（41.46）	*P*_1_＝0.392
单独IE-1	5	0（0）	*P*_1_＝0.168，*P*_2_＝0.152
PP65+IE-1	52	13（25.00）	*P*_1_＝0.228，*P*_2_＝0.064
PP65+PP50	5	3（60.00）	*P*_1_＝0.349，*P*_2_＝0.647
携带P65	139	51（36.69）	*P*_1_＝0.805，*P*_2_＝0.567
携带IE-1	57	13（22.81）	*P*_1_＝0.130，*P*_2_＝0.022

5. 不同供者HLA型别移植后严重CMV感染发生率：经病原学确诊，146例患者中发生CMV肺炎5例、CMV脑炎1例、CMV病（肺炎+脑炎+视网膜炎）1例。在PP65组发生CMV组织病4例（3例CMV肺炎，1例CMV肺炎+脑炎+视网膜炎），在伴PP65+IE-1组发生1例（CMV肠炎），PP65+PP50组2例（CMV肺炎2例）。R×C表格检验显示*P*<0.001，进一步进行组间比较，PP65组和携带IE-1组差异均无统计学意义，PP65+PP50组显示严重的CMV组织器官病发生率显著高于其他组，但该组只有5例病例，需要更多病例数验证此结果（表3）。

**表3:** 不同HLA型别组异基因造血干细胞移植后巨细胞病毒（CMV）组织器官病发生率

HLA型别分组	例数	CMV组织器官病［例（％）］	*P*值（*P*_1_：与总体比较；*P*_2_：与PP65组比较）
总体（所有患者）	146	7（0.05）	
PP65	82	4（0.05）	*P*_1_＝0.977
IE-1	5	0（0）	*P*_1_＝0.616，*P*_2_＝0.613
PP65+IE-1	52	1（0.02）	*P*_1_＝0.367，*P*_2_＝0.379
PP65+PP50	5	2（40.00）	*P*_1_＝0.001，*P*_2_＝0.003
携带PP65	139	7（0.05）	*P*_1_＝1.000，*P*_2_＝0.958
携带IE-1	57	1（0.02）	*P*_1_＝0.317，*P*_2_＝0.322

## 讨论

近年，随着移植方案的不断改进、单倍型移植越来越多地应用于临床，国内移植治疗患者例数和移植治疗适应证明显增加[6],[8]，移植后CMV感染导致各种感染综合征以及骨髓抑制等严重影响移植治疗效果和预后，也是GVHD和移植相关血栓性微血管病（TA-TMA）的重要危险因素之一，特别严重的CMV肺炎、CMV脑炎、CMV肠炎、CMV视网膜炎等可能直接导致患者死亡，因此有效的预防治疗和治疗方案（包括CMV特异性T细胞过继免疫治疗）一直是研究的热点[9]–[12]。

本组146例血液病患儿allo-HSCT后180 d CMV感染的发生率为34.93％（51/146），与文献[13]报道的30％～70％发生率相符，未见单倍型移植较全相合移植有更高的CMV激活风险，Bu-Cy+ATG预处理方案与PT-Cy+ATG预处理方案在CMV激活风险上也未见明显区别，该结果与史红鱼等[2]报告的结果一致。

PP65和IE-1均为CMV外壳的蛋白成分[14]，在CMV感染细胞和机体早期的抗CMV免疫中均具有关键性作用。已有研究表明不同HLA型别个体的免疫细胞对不同抗原肽刺激反应导致差异性的免疫效果和保护作用，其中HLA可能发挥了关键作用[4],[15]。CMV抗原表位与CTL结合力的高低与HLA等位基因密切相关，HLA等位基因识别位点的空间构象差异及与抗原肽的亲和力高低与抗病毒免疫有重要关系[16]。本研究结果显示，供者HLA型别与IE-1亲和力高，则患者具有较低的移植后CMV感染率和CMV组织器官病发生率，提示allo-HSCT患者供者来源造血重建过程中，CMV特异性免疫重建和CMV易感性及感染保护能力可能因供者HLA型别不同而有所差异。这一结果与Bestard等[17]在肾移植研究中发现移植前IE-1特异性T细胞数量低与移植后CMV感染相关类似，Shin等[18]在肝移植研究中也发现IE-1特异性T细胞阳性个体移植后2个月CMV感染率更低。Barabas等[19]研究发现，在健康人群中，虽然血清学阳性个体中PP65刺激后IFN-γ斑点细胞数量更多，但在血清学阴性个体中IE-1刺激后IFN-γ斑点细胞数更高，这可能部分解释了我们的研究结果，在初始的CMV免疫功能建立过程中，IE-1较PP65可能有更强（快）刺激机体产生CMV特异性CTL的能力，IE-1高亲和力HLA型别来源供者干细胞在移植后CMV特异性免疫重建过程中可能存在优势。

PP65和IE-1作为目前研究最多的两种抗原肽，其对应的高亲和力HLA等位基因型大部分已经确定，已有研究表明在实体器官移植患者中移植前用不同抗原肽刺激检测CTL数量和功能有不同的临床预测价值[17],[20]。本研究中，93.8％的供者T细胞均与PP65抗原肽具有高亲和力，而与IE-1高亲和力的供者在39.0％左右，联合应用PP65和IE-1抗原肽可以与99.8％的供者免疫细胞产生高亲和力免疫反应，这也证明了Barabas等[19]使用同时包含PP65和IE-1抗原肽作为刺激剂的合理性。

本研究结果提示，在allo-HSCT后的过继免疫或免疫重建中，供者PP65和IE-1识别模式非常重要，选择CMV IE-1高亲和力型别或可减低移植后CMV激活感染的风险。其次，在制备用于过继免疫的CMV特异性CTL时，同时应用PP65和IE-1抗原肽刺激可能得到抗病毒能力更强的CTL细胞。不同HLA型别免疫细胞用于过继免疫治疗时，应用不同抗原肽刺激及如何应用这些抗原肽刺激都是值得研究的方向，不同抗原肽刺激后的CMV特异性CTL应用于患者后的临床效果也需要进一步大样本研究来评价。在CMV特异性CTL检测研究中，应用不同的抗原肽来构建多聚体也需要考虑供者（受者）HLA型别在识别这些抗原肽间的差异。

综上所述，移植后CMV免疫重建及免疫保护尚有许多未明机制，免疫细胞的抗原识别提呈过程及产生有效的免疫保护细胞仍是关键，在儿童allo-HSCT中，供者HLA型别为IE-1高亲和力时，移植后可能有更低的CMV感染率。限于该研究观察例数的限制，需要进一步设计多中心前瞻性研究，将HLA型别与PP65、IE-1、CMV lysate刺激后CMV特异性CTL数量及产生时间进行探索，同时更多观察不同抗原肽刺激不同HLA型别供者淋巴细胞后的CMV特异性CTL过继免疫治疗临床效果来验证在临床应用的价值，为临床应用提供直接的指导意义。
